# Effects of behavioural interventions on postpartum retention and adherence among women with HIV on lifelong ART: the results of a cluster randomized trial in Kenya (the MOTIVATE trial)

**DOI:** 10.1002/jia2.25852

**Published:** 2022-01-18

**Authors:** Lisa L. Abuogi, Maricianah Onono, Thomas A. Odeny, Kevin Owuor, Anna Helova, Karen Hampanda, Tobias Odwar, Dickens Onyango, Leslie A. McClure, Elizabeth A. Bukusi, Janet M. Turan

**Affiliations:** ^1^ Department of Pediatrics University of Colorado Denver Aurora Colorado USA; ^2^ Center for Global Health Colorado School of Public Health University of Colorado Anschutz Medical Campus Aurora Colorado USA; ^3^ Centre for Microbiology Research Kenya Medical Research Institute Nairobi Kenya; ^4^ National Cancer Institute National Institutes of Health Bethesda Maryland USA; ^5^ Department of Biostatistics School of Public Health University of Alabama at Birmingham Birmingham Alabama USA; ^6^ Department of Health Care Policy and Organization School of Public Health University of Alabama at Birmingham Birmingham Alabama USA; ^7^ Department of Obstetrics and Gynecology University of Colorado Anschutz Medical Campus Aurora Colorado USA; ^8^ Department of Health Kisumu Kenya; ^9^ Department of Epidemiology and Biostatistics Dornsife School of Public Health Drexel University Philadelphia Pennsylvania USA

**Keywords:** women living with HIV, postpartum, retention, viral suppression, PMTCT

## Abstract

**Introduction:**

Retention in HIV care and adherence to antiretroviral therapy (ART) during pregnancy and postpartum for women living with HIV (WLWH) are necessary to optimize health outcomes for women and infants. The objective of this study was to evaluate the impact of two evidenced‐based behavioural interventions on postpartum adherence and retention in WLWH in Kenya.

**Methods:**

The Mother‐Infant Visit Adherence and Treatment Engagement (MOTIVATE) study was a cluster‐randomized trial enrolling pregnant WLWH from December 2015 to August 2017. Twenty‐four health facilities in southwestern Kenya were randomized to: (1) standard care (control), (2) text‐messaging, (3) community‐based mentor mothers (cMM) or (4) text‐messaging and cMM. Primary outcomes included retention in care and ART adherence at 12 months postpartum. Analyses utilized generalized estimating equations and competing risks regression. Per‐protocol analyses examined differences in postpartum retention for women with high versus low levels of exposure to the interventions.

**Results:**

We enrolled 1331 pregnant WLWH (mean age 28 years). At 12 months postpartum, 1140 (85.6%) women were retained in care, 96 women (7.2%) were lost‐to‐follow‐up (LTFU) and 95 (7.1%) were discontinued from the study. In intention‐to‐treat analyses, the relative risk of being retained at 12‐months postpartum was not significantly higher in the intervention arms versus the control arm. In time‐to‐event analysis, the cMM and text arm had significantly lower rates of LTFU (hazard ratio 0.44, *p* = 0.019). In per‐protocol analysis, the relative risk of 12‐month postpartum retention was 24–29% higher for women receiving at least 80% of the expected intervention compared to the control arm; text message only risk ratio (RR) 1.24 (95% confidence interval [CI] 1.16–1.32, *p*<0.001), cMM only RR 1.29 (95% CI 1.21–1.37, *p*<0.001) and cMM plus text RR 1.29 (1.21–1.37, *p*<0.001). Women LTFU were younger (*p*<0.001), less likely to be married (*p*<0.001) and more likely to be newly diagnosed with HIV during pregnancy (*p*<0.001). Self‐reported ART adherence did not vary by study arm.

**Conclusions:**

Behavioural interventions using peer support and text messages did not appear to improve 12‐month postpartum retention and adherence in intention‐to‐treat analyses. Higher levels of exposure to the interventions may be necessary to achieve the desired effects.

## 1 INTRODUCTION

In 2019, an estimated 85% of pregnant women living with HIV (WLWH) globally had access to lifelong antiretroviral therapy (ART) for the prevention of mother‐to‐child transmission (PMTCT) [1]. Yet, lack of retention of pregnant/postpartum women living with HIV (PWLWH) in HIV care throughout the peripartum period and sub‐optimal adherence to ART continue to undermine the health of WLWH and increase the risk of HIV transmission to infants and uninfected partners [2, 3, 4, 5].

Behavioural interventions to improve retention and adherence on lifelong ART for PWLWH in lower resource settings have shown mixed results and lacked rigorous, randomized study designs [4, 6, 7]. However, encouraging results support the potential of mobile health (mHealth) and peer support approaches [8, 9]. Early studies have generally shown high acceptability of text messaging among PWLWH in sub‐Saharan Africa (SSA) and the potential to improve uptake of PMTCT services, clinic attendance and medication adherence [10, 11, 12, 13, 14]. Lay health workers, including mentor mothers, who provide psychosocial support to other PWLWH, have been utilized widely in SSA with improvements in uptake of PMTCT services, but have not been rigorously evaluated [15, 16, 17, 18].

Aimed at addressing the need to improve engagement in HIV care and adherence to ART during the pregnancy and postpartum periods, the Mother‐Infant Visit Adherence and Treatment Engagement (MOTIVATE) study was launched shortly after Kenya's rollout of lifelong ART for all PWLWH in 2015. The study aimed to evaluate the impact of two evidenced‐based behavioural interventions (text messaging and home visits by community mentor mothers [cMM]) on retention in HIV care and ART adherence at 12 months postpartum for PWLWH.

## 2 METHODS

The MOTIVATE study was a cluster‐randomized, factorial, controlled trial [19]. Twenty‐four Kenyan Ministry of Health facilities were randomized to one of four study arms: (1) standard care (control), (2) text‐messaging only, (3) cMM only or (4) both text‐messaging and cMM.

### 2.1 Setting

The study was conducted in southwestern Kenyan in Migori, Kisumu and Homabay Counties with adult HIV prevalence ranging from 13.0% to 19.6%, compared to 4.9% nationally [20]. Kenya adopted universal lifelong ART for PWLWH in 2015 [21, 22]. All of the 24 government healthcare facilities participating in the study provided free PMTCT services, including ART (dolutegravir not available), integrated into antenatal and postnatal care through 18 months postpartum along with HIV infant testing and follow up. Clinic visits and ART refills occurred every 1–3 months per Kenyan guidelines and differentiated service delivery models were not in effect. Infant HIV testing occurred at 6 weeks, 6 months and 12 months using HIV PCR and at 18 months using antibody testing. Facilities were purposively selected to represent a distribution of urban and rural, high and lower volume, and geographic locations (e.g. sub‐county). Twenty sites were initially chosen, and four additional sites were added to achieve target enrolment.

### 2.2 Participants

PWLWH were recruited during routine antenatal clinic visits December 2015 to August 2017 and followed through 1‐year postpartum (March 2019). Inclusion criteria included: women with HIV of at least 18 years of age, mobile phone access (with disclosure of their HIV status to any person sharing the phone), willingness to have home visits (or meetings with a cMM in alternate location) and living within the facility catchment area. After enrolment, women were discontinued from the study if they experienced a miscarriage, stillbirth or other child loss as the study interventions were designed for mother–child pairs.

### 2.3 Study interventions

The study interventions and their development during the pre‐trial formative phase have been described in detail elsewhere [11, 13, 19, 23]. In brief, the text messaging intervention was developed based on qualitative formative research and the Health Belief Model [24, 25]. Participants received free tailored text messages delivered by automated texting software. Message content focused on medication and clinic adherence without explicit mention of HIV or ART as well as promotion of maternal and child healthcare services timed to the stage of pregnancy and age of the infant post‐delivery. Messages were sent weekly from study enrolment in pregnancy through 12 months postpartum. Participants were also able to engage by phone without charge with a study nurse. Study staff continuously reviewed texting software logs of all messages sent, delivered (or failed) and received throughout the study.

The cMM intervention was adapted from the Kenya Mentor Mother Program and has been previously described [23, 26]. cMMs were WLWH on ART with a recent pregnancy, with a minimum of 8 years of primary education, who had demonstrated good ART adherence and who had disclosed their HIV status. In contrast to traditional mentor mothers who are based at health facilities, cMMs in this study worked from the community and carried out structured home visits. The cMM visit schedule included up to four antenatal visits and nine post‐natal visits designed to maximize retention in PMTCT and other essential health services. cMMs were supervised by study nurses and underwent observed home visits, regular refresher trainings, quarterly evaluations and review of visit logs.

### 2.4 Randomization

Randomization occurred at the facility (cluster) level given concerns for contamination if interventions delivered at the individual level. A participatory open community randomization meeting was held at which a study facility representative chose a sealed opaque envelope with their site's randomization arm. Facilities were stratified by geographic region and each region had at least one site in each of the four study arms.

### 2.5 Data collection and measures

All study data were abstracted by trained data clerks from standardized Kenyan Ministry of Health HIV clinic visit forms and clinic registers (i.e. medical records) at the study facilities into an Open Data Kit (ODK) electronic database with built‐in quality controls.

Primary outcome measures were adherence to ART and retention in HIV care at 12 months post‐partum. ART adherence was measured via self‐report, recorded in the woman's medical record during routine visits. Adherence was classified as per Kenya guidelines as good (>95% of pills taken since last clinic visit), fair (85–94%) or poor (<85%). Given >95% of women reported good adherence throughout the study, this was compared versus combined fair or poor adherence. Secondary outcomes included viral suppression and infant outcomes. Viral load was added as a secondary measurement of adherence after routine viral load monitoring was introduced in Kenya after the study launched, and was obtained through health records. Per the Kenya National ART guidelines, viral load was obtained by clinic providers after 6 months of ART for those newly initiating (or at time of pregnancy identification for those already on ART) and every 6 months until cessation of breastfeeding [22]. Those with viral load <1000 copies/ml were considered virally suppressed. Retention in care at 12 months postpartum was defined as the proportion of women who had an HIV care visit within 90 days at 12 months after delivery. Women with documented transfer to another clinic, which required an official transfer letter, were considered retained in care. Women who died, experienced a pregnancy loss or child death, or withdrew were discontinued from the study and their data were used up until study discontinuation.

### 2.6 Sample size

The study was powered based on estimated differences in proportions of maternal ART adherence and retention in the intervention versus the control arms at 12 months postpartum accounting for clustering as previously reported [19, 27]. We estimated a baseline ART adherence rate of 55% based on pre‐universal ART rates found in the literature for pregnant women in SSA at the time of study design [28] and a baseline retention in care rate of 48% based on trial data from a study of service integration conducted in the same region in Kenya [29]. We powered our study to be able to detect a 25% absolute increase due to each intervention individually. To achieve 80% power for both outcomes with a conservative (high) intra‐cluster correlation coefficient of 0.12 and an average of 70 women sampled per community, we calculated 20 sites, 5 in each arm with a target sample size of 1336 women (334 women per arm) would be needed.

### 2.7 Analysis

Descriptive analyses summarized characteristics of participants and assessed frequency distributions of variables. Analyses of primary outcomes of maternal ART adherence and maternal retention in care (dichotomous variables) compared proportions between intervention and control groups using cluster adjusted two‐sided chi square tests. Given the clustered study design, generalized estimating equations (GEEs) were used to test for differences of interest in univariable and multivariable models, adjusted for relevant covariates identified as differing across study arms at baseline with *p*≤0.05, using a logit link. Secondary outcomes, such as maternal viral load suppression (<1000 copies/ml), were analysed using similar methods, considering the nature of the variable (continuous, dichotomous or time to event). Time to LTFU (defined as no clinic visit ± 90 days at 12 months postpartum) was modelled using competing risks regression analysis accounting for clustering using GEE with resulting in cause‐specific hazard ratios (HRs) and displayed using cumulative incidence plot. Participant or infant/child death or pregnancy loss, as well as transfers out were considered competing events for the LTFU and retention outcomes and were censored at the time of the event. Results were summarized as risk ratios, HRs and mean changes, as appropriate; respective 95% confidence intervals are reported based on the robust variance estimates from the GEE models.

Primary analyses were intention‐to‐treat (ITT), while secondary per‐protocol (PP) analyses examined differences in outcomes for women with high (>80% of expected intervention dose received) versus low levels of exposure to the interventions, adjusted for gestational age at enrolment to the study. For the PP analyses, a level of completion of 80% of the intervention received was selected a priori as this was considered by the research team to be the minimal adequate dose of the interventions. Although there is no standard recommended cut‐off in the literature for the dose of multi‐session interventions received to be used in PP analyses, “clinical” judgement of an adequate dose is one accepted approach [30]. All analyses were done using Stata 15 Software [31].

### 2.8 Ethics

All women enrolled in the study provided written informed consent. The study was approved by the Kenya Medical Research Institute, University of Alabama at Birmingham and the University of Colorado, Denver Institutional Review Boards.

## 3 RESULTS

**Figure 1:**
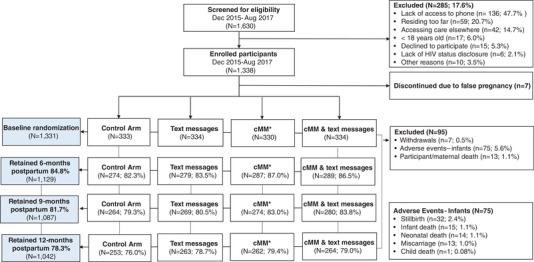
MOTIVATE study population.

A total of 1338 WLWH were enrolled to the study, of whom seven were subsequently discontinued because they were not pregnant (Figure 1). The mean age of the remaining 1331 was 28 years (standard deviation 6.0), with a median gestational age of 24 weeks at study enrolment (interquartile range [IQR] 20–30) (Table 1). Nearly, 80% knew their HIV diagnosis prior to the index pregnancy. Only 8% of women overall were primigravida at study enrolment, which varied somewhat (*p* = 0.05) by study arm (Table 1). All women had been initiated on ART and median time on ART at enrolment was 18 months (IQR 2–40). For women with baseline self‐report of ART adherence available, 1063 (96.8%) reported good adherence, 35 (3.2%) fair or poor adherence.

**Table 1:** Baseline characteristics of pregnant women living with HIV on ART enrolling into the MOTIVATE study by study arm

**Baseline variables**	**Control**	**Text message only**	**CMM only**	**CMM and text message**	**Total**	
Total	***N* = 333 (%)**	***N* = 334 (%)**	***N* = 330 (%)**	***N* = 334 (%)**	***N* = 1331 (%)**	***p* Value**
Enrolment age years						0.839
Mean (SD)	28.8 (5.5)	28.0 (5.4)	28.3 (5.5)	28.9 (5.8)	28.5 (5.6)	
Enrolment age (years)						0.978
<25 years	80 (24.0)	101 (30.2)	92 (27.9)	88 (26.3)	361 (27.1)	
25–28 years	86 (25.8)	77 (23.1)	76 (23.0)	78 (23.4)	317 (23.8)	
29–32 years	83 (24.9)	84 (25.1)	85 (25.8)	78 (23.4)	330 (24.8)	
33+ years	84 (25.2)	72 (21.6)	77 (23.3)	90 (26.9)	323 (24.3)	
Marital status						0.640
Married	308 (92.5)	306 (91.6)	287 (87.0)	317 (94.9)	1218 (91.5)	
Widowed	9 (2.7)	11 (3.3)	12 (3.6)	4 (1.2)	36 (2.7)	
Single	14 (4.2)	13 (3.9)	25 (7.6)	10 (3.0)	62 (4.7)	
Divorce	1 (0.3)	1 (0.3)	1 (0.3)	2 (0.6)	5 (0.4)	
Separated	1 (0.3)	3 (0.9)	5 (1.5)	1 (0.3)	10 (0.8)	
Gestational period (weeks)						0.143
Median (IQR)	26.0 (20.0–30.0)	26.0 (20.0–30.0)	24.0 (19.0–28.0)	24.0 (16.0–28.0)	24.0 (20.0–30.0)	
Gravida						0.050
1	31 (9.3)	26 (7.8)	34 (10.3)	16 (4.8)	107 (8.0)	
≥2	302 (90.7)	308 (92.2)	296 (89.7)	318 (95.2)	1224 (92.0)	
HIV status at time of pregnancy						0.122
Known positive	257 (77.2)	248 (74.3)	251 (76.1)	293 (87.7)	1049 (78.8)	
New diagnosis	76 (22.8)	86 (25.7)	79 (23.9)	41 (12.3)	282 (21.2)	
ART regimen						0.475
PI‐based	13 (3.9)	23 (6.9)	11 (3.3)	14 (4.2)	61 (4.6)	
NNRTI‐based	320 (96.1)	311 (93.1)	319 (96.7)	320 (95.8)	1270 (95.4)	
Baseline ART adherence^a^						0.695
Fair/poor (*n* = 35)	14 (4.2)	7 (2.1)	8 (2.4)	6 (1.8)	35 (2.6)	
Good (*n* = 1063)	267 (80.2)	256 (76.6)	246 (74.5)	294 (88.0)	1063 (79.9)	
Missing	52 (15.6)	71 (21.3)	76 (23.0)	34 (10.2)	233 (17.5)	
Time on ART (months)						0.073
Median (IQR)	17.0 (3.0–41.0)	11.0 (1.0–33.0)	17.0 (2.0–35.0)	29.0 (9.0–50.0)	18.0 (2.0–40.0)	
Baseline CD4 count^a^						0.824
Median (IQR)	520.0 (383.5–637.5)	480.5 (270.5–721.5)	499.0 (369.0–648.0)	536.0 (391.0–674.0)	516.5 (348.5–667.5)	
Baseline viral load^a^						0.900
Undetectable	148 (44.4)	150 (44.9)	148 (44.8)	172 (51.5)	618 (46.4)	
<1000 copies^b^	55 (16.5)	22 (6.6)	31 (9.4)	26 (7.8)	134 (10.1)	
≥1000 copies	20 (6.0)	19 (5.7)	16 (4.8)	15 (4.5)	70 (5.3)	
Missing	110 (33.0)	143 (42.8)	135 (40.9)	121 (36.2)	509 (38.2)	
Baseline WHO Stage						0.985
WHO Stage I	213 (64.0)	223 (66.8)	198 (60.0)	182 (54.5)	816 (61.3)	
WHO Stage II	82 (24.6)	75 (22.5)	97 (29.4)	112 (33.5)	366 (27.5)	
WHO Stage III	31 (9.3)	31 (9.3)	32 (9.7)	35 (10.5)	129 (9.7)	
WHO Stage IV	6 (1.8)	5 (1.5)	3 (0.9)	5 (1.5)	19 (1.4)	

Abbreviations: ART, antiretroviral treatment; CMM, community mentor mother; IQR, interquartile range; NNRTI, non‐nucleoside reverse transcriptase inhibitor; PI, protease inhibitor; SD, standard deviation; WHO, World Health Organization.

^a^ Within 3 months of study enrolment.

^b^Does not include undetectable viral loads.

### 3.1 Postpartum retention in HIV care

At 12 months postpartum, 1140 (85.6%) women were retained in care, including 98 (7.4%) who transferred care to another clinic. Ninety‐six (7.2%) women were LTFU, 7 (0.5%) withdrew and 13 (1.0%) died. Additionally, 75 women (5.6%) experienced pregnancy loss (13 miscarriages and 32 stillbirths) or other child death (14 neonatal deaths, 15 infant deaths and 1 child death) and were discontinued prior to 12 months postpartum. Retention was highest in Kisumu county (84%), followed by Migori (77%) and lowest in Homa Bay (72%), *p* = 0.013. The adjusted risk of being retained at 12 months postpartum (adjusted for gravidity and time on ART) was not significantly higher in the intervention arms versus control arm (Table 2).

**Table 2:** Comparison of retention and adherence at 12 months postpartum by study arm

Outcome	*N*	*N* (%)	Risk ratio (95% confidence interval)	*p* Value
Retention^a^	1331			
Control		253 (76.0)	Reference	
Text message only		263 (78.7)	1.08 (0.97–1.21)	0.141
CMM only		262 (79.4)	1.06 (0.96–1.17)	0.248
CMM and text message		264 (79.0)	1.04 (0.94–1.15)	0.460
Self‐reported ART good adherence^b^	1041			
Control		247 (97.6)	Reference	
Text message only		258 (98.5)	1.01 (0.99–1.03)	0.387
CMM only		253 (96.6)	0.99 (0.95–1.03)	0.554
CMM and text message		255 (96.6)	0.99 (0.94–1.04)	0.664

Abbreviations: ART, antiretroviral treatment; CMM, community mentor mother.

^a^ Adjusted for age and gravidity.

^b^ Not adjusted for age and gravidity due to nonconvergence.

**Figure 2:**
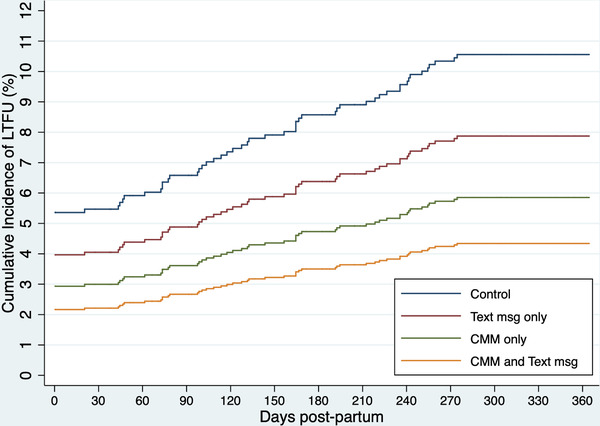
Competing risks regression model of cumulative incidence of proportion of women lost to follow up from delivery to 12 months postpartum by study arm.

Overall, women who were LTFU were younger (*p*<0.001), less likely to be married (*p*<0.001), more likely to be newly diagnosed with HIV at start of pregnancy (*p*<0.001) and to report sub‐optimal ART adherence at study enrolment (*p*<0.001; Table 3). Viral suppression at baseline was not associated with LTFU. Figure 2 displays the cumulative incidence of LTFU from delivery through 12 months postpartum by study arm. Women in the control group consistently had the highest cumulative incidence of LTFU over time. All intervention arms had consistently lower incidence of postpartum LTFU, but only the combined cMM and text message group was statistically significantly different from the control (HR: 0.44, 95% CI 0.22–0.88; *p* = 0.019). Sub‐group analyses comparing women <24 years of age to women ≥ 24 years of age and women with known versus new diagnosis of HIV at start of pregnancy did not show differential affects by study arm (data not shown).

**Table 3:** Comparison of baseline characteristics by HIV care engagement and viral load outcomes at 12 months postpartum

	Retention in care					Viral suppression		
	Total *n* = 1331	Retained (*n* = 1235)	Lost to follow up (*n* = 96)	*p* Value	Total *n* = 791	>1000 copies/ml (*n* = 43)	≤1000 copies/ml (*n* = 748)	*p* Value
Enrolment age (years)				<0.001				0.7429
<25	361	316 (25.6)	45 (46.9)		201	11 (25.6)	190 (25.4)	
25–28	317	292 (23.6)	25 (26.0)		184	8 (18.6)	176 (23.5)	
29–32	330	310 (25.1)	20 (20.8)		209	14 (32.6)	195 (26.1)	
≥33	323	317 (25.7)	6 (6.2)		197	10 (23.3)	187 (25.0)	
Marital status				0.011				0.933
Married	1218	1134 (91.8)	84 (87.5)		734	40 (93.0)	694 (92.8)	
Not married	113	101 (8.2)	12 (12.5)		57	3 (7.0)	54 (7.2)	
Gravidity				0.016				0.016
1	107	93 (7.5)	14 (14.6)		53	3 (7.0)	50 (6.7)	
≥2	1224	1142 (92.5)	82 (85.4)		738	40 (93.0)	698 (93.3)	
HIV status at pregnancy				<0.001				0.692
Known positive	1049	1004 (81.3)	45 (46.9)		630	35 (81.4)	595 (79.5)	
Newly positive	282	231 (18.7)	51 (53.1)		161	8 (18.6)	153 (20.5)	
ART regimen				0.229				0.372
PI‐based	61	59 (4.8)	2 (2.1)		38	3 (7.0)	35 (4.7)	
NNRTI‐based	1270	1176 (95.2)	94 (97.9)		753	40 (93.0)	713 (95.3)	
Baseline adherence				<0.001				0.040
Fair/poor	35	29 (2.4)	6 (6.3)		20	3 (7.0)	17 (2.3)	
Good	1063	1019 (82.5)	44 (46.3)		648	31 (72.1)	617 (82.5)	
Missing	232	187 (15.1)	45 (47.4)		123	9 (20.9)	114 (15.2)	
Baseline viral load	1128			0.763				<0.001
< 1000 copies/ml	999	957 (88.6)	42 (87.5)		614	18 (50.0)	596 (91.6)	
≥1000 copies/ml	129	123 (11.4)	6 (12.5)		73	18 (50.0)	55 (8.4)	

Abbreviations: NNRTI, non‐nucleoside reverse transcriptase inhibitor; PI, protease inhibitor.

### 3.2 Adherence

Self‐reported ART adherence at 12 months postpartum was available for 1042 women (all of those retained) of which 1013 (97.2%) reported overall good adherence with only 29 (2.8%) women reporting fair or poor adherence. The adjusted risk ratios for good versus fair/poor adherence at 12 months postpartum were not statistically different between intervention arms and the control arm (Table 2).

In the secondary analysis of viral load suppression, among 791 (59.4%) women with a viral load result at 12 months postpartum, only 43 (5.4%) were unsuppressed and these women more often reported sub‐optimal adherence at study enrolment (*p* = 0.040) and had a history of prior high viral load (*p*<0.001).

### 3.3 Infant outcomes

A total of 1156 (87.1%) women had a live birth after study enrolment resulting in 1167 infants; 1156 (99.1%) infants underwent HIV testing. At 12 months of age, seven (0.6%) of tested infants were diagnosed with HIV, which did not differ by study arm (*p* = 0.719) (Table S1).

### 3.4 Intervention fidelity and PP analysis

Women in the cMM arms (*n* = 664) received a median of 11 cMM visits (IQR 8–12) out of possible 13 total visits and 358 women (54.0%) received at least 80% of intended visits. Among 666 women randomized to the text messaging intervention, women received a median of 51 texts (IQR 16–60) out of a median expected 64 (IQR 57–71) texts based on gestational age at study enrolment. A total of 307 (46.0%) received at least 80% of expected texts. Baseline characteristics were not statistically different among women who received 80% or more of the interventions and those who did not (data not shown), nor were they different as compared to the total cohort overall (Table S2).

In PP analysis comparing intervention participants who received at least 80% of the expected intervention to those in control, the relative risk of 12‐month postpartum retention was significantly higher in all intervention arms as compared to the control arm (*p*<0.001) (Table 4).

**Table 4:** Per‐protocol analysis comparing retention at 12 months postpartum by study arm in women who received at least 80% of the expected study intervention

Outcome Retention	Overall retention *N* = 1331 *N* (%)	Retention if ≥80% of intervention received *N* = 745 *N* (%)	Risk ratio (95% confidence interval)	*p* Value
Control	253 (76.0)	253 (76.0)	Reference	
Text message only	263 (78.7)	138 (93.9)	1.24 (1.16–1.32)	<0.001
CMM only	262 (79.4)	165 (97.6)	1.29 (1.21–1.37)	<0.001
CMM and text message	264 (79.0)	94 (97.9)	1.29 (1.21–1.37)	<0.001

Abbreviation: CMM, community mentor mother.

## 4 DISCUSSION

This study examined the individual and combined effects of two behavioural interventions (text messaging and cMMs) to promote retention and adherence among PWLWH on ART through a large cluster randomized prospective trial in southwestern Kenya. In ITT analysis, 12‐month postpartum retention and ART adherence among PWLWH receiving our behavioural interventions was not significantly different than in PWLWH in the control arm. However, in PP analysis among women who received at least 80% of the expected intervention, the relative risk of 12‐month postpartum retention was 24–29% higher for women receiving an intervention. Further, sub‐analysis demonstrated that the combination of peer support from cMMs and text messages reduced the risk of LTFU compared to the control by more than 50% throughout the postpartum period.

While ITT analysis of primary study outcomes did not show increased postpartum retention at 12 months, PP analysis demonstrated significant and increasing impact of text messages alone, followed by cMM visits alone and cMM and text messaging combined, if women received at least 80% of the expected intervention. It is important to note that, despite rigorous study procedures to support intervention fidelity, only 54% of women in the cMM arms and 46% in the text arms received 80% or more of the expected intervention. The incomplete receipt of interventions by a significant proportion of women likely lessened the impact of these interventions and made women in the intervention arms appear more like women in the control arm, thus making it more difficult to identify differences between arms in ITT analysis. While baseline characteristics did not differ, it is possible that unmeasured variables were predictive of women more likely to receive the interventions. During interviews with cMMs, they expressed challenges delivering home visits immediately after delivery and when disclosure to household members was lacking, as well as difficulties related to weather and distance (manuscript in press). For women receiving text messages, while mobile phone access is high in Kenya, it can be precarious, especially for women who may need to accommodate phone sharing with others, interfering with consistent delivery of our text intervention [32, 33]. Additionally, we excluded women without phones or with shared phones who had not disclosed, which may have biased our sample to less vulnerable women who were less likely to benefit from the interventions. Additionally, while intervention satisfaction among retained women was high (unpublished data), it is possible that women LTFU engaged less with the interventions and were less satisfied with them. We hypothesize that women with consistent phone access and those able to receive regular mentor mother visits (at home or elsewhere in the community) will benefit most from these interventions if implemented. Thus, although our PP analyses had promising results for women who received higher exposure to the interventions, the potential biases and limitations of PP analyses are acknowledged [34].

Other studies aimed at improving PMTCT service uptake and retention across the HIV care continuum in SSA have shown mixed results [6, 7, 8, 35]. We hypothesize that higher rates of adherence and retention found in our study differ from findings in other settings from mostly retrospective cohort studies may relate to different populations of women, less established country‐level ART programs and failure to account for transfer outs and pregnancy losses [8, 36, 37, 38, 39, 40]. Systematic reviews have found that the evidence base for interventions to improve postpartum retention is overall weak [35], and few studies address the entire peripartum period or demonstrate intervention effectiveness post‐universal ART [7, 29, 35]. More recently, only two of six INtegrating and Scaling up PMTCT Through Implementation REsearch (INSPIRE) collaboration studies to improve retention of PWLWH on ART showed a significant effect [41]. One, a non‐randomized study of facility mentor mothers in Nigeria, and the other, a cluster randomized study in Malawi, showed benefit of peer‐based patient support both at the facility and community levels on service uptake and 2‐year retention in care [15, 42]. Similarly, a systematic review of community‐based versus facility‐based interventions for improving PMTCT‐related HIV outcomes showed some benefits [43]. In contrast, several text‐based approaches to improve PMTCT outcomes have not shown efficacy [10, 44].

Strengths of this study include the prospective, cluster‐randomized design to minimize confounding and the risk of contamination of the interventions, and the use of theory‐based interventions developed with input from key stakeholders. Additionally, our study findings are likely to be externally valid and generalizable to other HIV programs in SSA. However, while this was a large‐scale, rigorously conducted study, several limitations exist. Our primary ART adherence outcome relied on self‐reported adherence, which is known to be less accurate than other measures of adherence, such as biomedical or pharmacologic methods [45, 46]. This outcome was chosen at the time of study conception as it was universally obtained in a standard manner at all clinic visits. Viral load monitoring, a more objective measurement of adherence, was newly introduced in Kenya during the study period, resulting in wide variation in viral load tests available and substantial missing data, limiting our interpretation of the impact of the interventions on viral suppression. Additionally, because we based our power estimates on the much lower pre‐universal ART estimates of retention and adherence in our study population, our sample size estimates underestimated the baseline levels of adherence and retention and overestimated potential effect sizes for our primary outcomes. Using a cluster randomized study design also presented some challenges, including the need for many clusters (facilities), which may have compromised the ability to deliver the interventions with high fidelity. An individually randomized design might have been able to achieve an appropriately powered sample size in a more efficient manner, but a cluster design was chosen due to concern for contamination between study arms if all interventions were available in each facility. Additionally, our interventions were not designed to specifically address important psychosocial risk factors among PWLWH, including mental health, stigma and intimate partner violence; nor did we capture data on these psychosocial factors limiting our ability to analyse their potential impact [47, 48, 49]. Finally, we utilized routinely collected program data, which were complete for many outcome variables (e.g. adherence) but not for viral load testing as discussed.

## 5 CONCLUSIONS

Overall, we did not find improved postpartum adherence and retention in women receiving text messaging and cMM support in ITT analyses. However, we did find when delivered at sufficient levels, these interventions may be able to significantly improve retention in HIV care at 1‐year postpartum. Integration of supportive interventions, such as text messaging and home visits by mentor mothers, may contribute to achieving the elimination of perinatal transmission of HIV and ensuring optimal health for WLWH and their infants but would benefit from further evaluation among PWLWH with higher risk of poor outcomes [50].

## COMPETING INTERESTS

None of the authors have competing interests to declare.

## AUTHORS’ CONTRIBUTIONS

LLA, MO, JMT, TAO, KO and EAB conceptualized, designed and conducted the study; analysed and interpreted the data; and drafted the manuscript. TO and DO contributed to study implementation, data collection and interpretation and manuscript writing. LAM provided statistical oversight, data analysis and manuscript review. AH and KH made substantial contributions to interpretation of the results and drafting of the manuscript. All authors read and approved the final manuscript.

## FUNDING

The MOTIVATE study was supported by Award Number 5R01HD080477 from the National Institute of Child Health and Human Development.

## DISCLAIMER

The content is solely the responsibility of the authors and does not necessarily represent the official views of the National Institute of Child Health and Human Development or the National Institutes of Health. The funders had no role in study design, data collection and analysis, decision to publish or preparation of the manuscript.

## Supporting information


**Table S1**. Description of infants diagnosed with HIV by mother's baseline characteristics and study arm

**Table S2**. Baseline characteristics of women received at least 80% of the intended interventions compared to those who received less than 80% of the interventions

## Data Availability

The data that support the findings of this study are available from the corresponding author upon reasonable request. The reasonable request would then need to be accompanied by the data use agreement signed by the University of Colorado, Denver.
