# Supplementary material for: Effects of behavioural interventions on postpartum retention and adherence among women with HIV on lifelong ART: the results of a cluster randomized trial in Kenya (the MOTIVATE trial)
Source: J Int AIDS Soc. 2022 Jan 18;25(1):e25852. doi: 10.1002/jia2.25852 (PMC8765560; doi:10.1002/jia2.25852)
Supplement: Supplementary file 1 — Table S1. Description of infants diagnosed with HIV by mother's baseline characteristics and study arm [file JIA2-25-e25852-s002.docx]

**Supplemental Table 1. Description of infants diagnosed with HIV by mother’s baseline characteristics and study arm**

|  | **Mother’s characteristics/study arm** | | | | | | | |
| --- | --- | --- | --- | --- | --- | --- | --- | --- |
| Infants diagnosed with HIV (N=7) | Study arm | >80% intervention dose | Enrolment age (yr) | Gravidity | New HIV diagnosis in pregnancy | ART regimen | Baseline adherence | Baseline viral load > 1000 copies/ml |
| **Infant 1** | Text | No | <25 | >2 | Yes | NNRTI-based | Good | Yes |
| **Infant 2** | Text | Yes | 29-32 | >2 | No | NNRTI-based | Good | Yes |
| **Infant 3** | cMM+text | No | <25 | >2 | No | NNRTI-based | Fair/Poor | Yes |
| **Infant 4** | CMM | No | 29-32 | >2 | No | NNRTI-based | Missing | No |
| **Infant 5** | Text | Yes | >33 | >2 | No | PI-based | Good | No |
| **Infant 6** | CMM | Yes | <25 | >2 | No | NNRTI-based | Good | No |
| **Infant 7** | CMM | No | 25-28 | >2 | No | NNRTI-based | Good | Yes |

CMM-community mentor mother, NNRTI-non-nucleoside reverse transcriptase inhibitor, PI-protease inhibitor
