# Supplementary material for: Effects of behavioural interventions on postpartum retention and adherence among women with HIV on lifelong ART: the results of a cluster randomized trial in Kenya (the MOTIVATE trial)
Source: J Int AIDS Soc. 2022 Jan 18;25(1):e25852. doi: 10.1002/jia2.25852 (PMC8765560; doi:10.1002/jia2.25852)
Supplement: Supplementary file 2 — Table S2. Baseline characteristics of women received at least 80% of the intended interventions compared to those who received less than 80% of the interventions [file JIA2-25-e25852-s001.docx]

| **Supplemental Table 2. Baseline characteristics of women received at least 80% of the intended interventions compared to those who received less than 80% of the interventions.** | | | | |
| --- | --- | --- | --- | --- |
| **Baseline Variables**  **N(%)** | **Partial Intervention Group**  **(<80% intervention)** | **Per Protocol Group**  **(> 80% intervention)** | **Total Intervention Participants** | **P-value** |
|  | **N=586 (58.7)** | **N=412 (41.3)** | **N=998 (100.0)** |  |
| Enrolment Age (years) |  |  |  | 0.90 |
| <25 years | 160 (27.3) | 121 (29.4) | 281 (28.2) |  |
| 25-28 years | 139 (23.7) | 92 (22.3) | 231 (23.1) |  |
| 29-32 years | 146 (24.9) | 101 (24.5) | 247 (24.7) |  |
| 33+ years | 141 (24.1) | 98 (23.8) | 239 (23.9) |  |
|  |  |  |  |  |
| Enrolment Age (years)  mean (SD) | 28.4 (5.6) | 28.4 (5.6) | 28.4 (5.6) | 0.89 |
|  |  |  |  |  |
| Marital Status |  |  |  | 0.45 |
| Married | 531 (90.6) | 379 (92.0) | 910 (91.2) |  |
| Not married | 55 (9.4) | 33 (8.0) | 88 (8.8) |  |
|  |  |  |  |  |
| HIV status at pregnancy |  |  |  | 0.26 |
| Known Positive | 458 (78.2) | 334 (81.1) | 792 (79.4) |  |
| Newly Diagnosis | 128 (21.8) | 78 (18.9) | 206 (20.6) |  |
|  |  |  |  |  |
| ART regimen |  |  |  | 0.51 |
| Protease Inhibitor-based | 26 (4.4) | 22 (5.3) | 48 (4.8) |  |
| NNRTI-based | 560 (95.6) | 390 (94.7) | 950 (95.2) |  |
|  |  |  |  |  |
| Gestational age (weeks)  mean (SD) | 24.2 (8.0) | 23.7 (7.3) | 24.0 (7.7) | 0.32 |
|  |  |  |  |  |
| Time on ART (months)  mean (SD) | 26.5 (27.7) | 24.6 (25.0) | 25.7 (26.6) | 0.25 |
|  |  |  |  |  |
| Baseline Adherence |  |  |  | 0.78 |
| Fair/Poor | 13 (2.7) | 8 (2.4) | 21 (2.6) |  |
| Good | 469 (97.3) | 327 (97.6) | 796 (97.4) |  |
|  |  |  |  |  |
| Gravida |  |  |  | 0.88 |
| <1 | 44 (7.5) | 32 (7.8) | 76 (7.6) |  |
| 2+ | 542 (92.5) | 380 (92.2) | 922 (92.4) |  |
|  |  |  |  |  |
| Gravida, mean (SD) | 3.5 (1.7) | 3.4 (1.6) | 3.5 (1.7) | 0.41 |
|  |  |  |  |  |
| Baseline viral load |  |  |  | 0.95 |
| <999 copies | 431 (88.5) | 328 (88.6) | 759 (88.6) |  |
| >1000 copies | 56 (11.5) | 42 (11.4) | 98 (11.4) |  |
|  |  |  |  |  |

NNRTI-non-nucleoside reverse transcriptase inhibitor, SD- standard deviation
